# Supplementary material for: PKHD1L1 is a coat protein of hair-cell stereocilia and is required for normal hearing
Source: Nat Commun. 2019 Aug 23;10:3801. doi: 10.1038/s41467-019-11712-w (PMC6707252; doi:10.1038/s41467-019-11712-w)
Supplement: Supplementary file 1 — Supplementary Information [file 41467_2019_11712_MOESM1_ESM.pdf]

Supplementary Information for

PKHD1L1 is a coat protein of hair-cell stereocilia and is required for normal hearing

Xudong Wu, Maryna V. Ivanchenko, Hoor Al Jandal, Marcelo Cicconet, Artur A. Indzhykulian & David P. Corey

Supplemental Figures

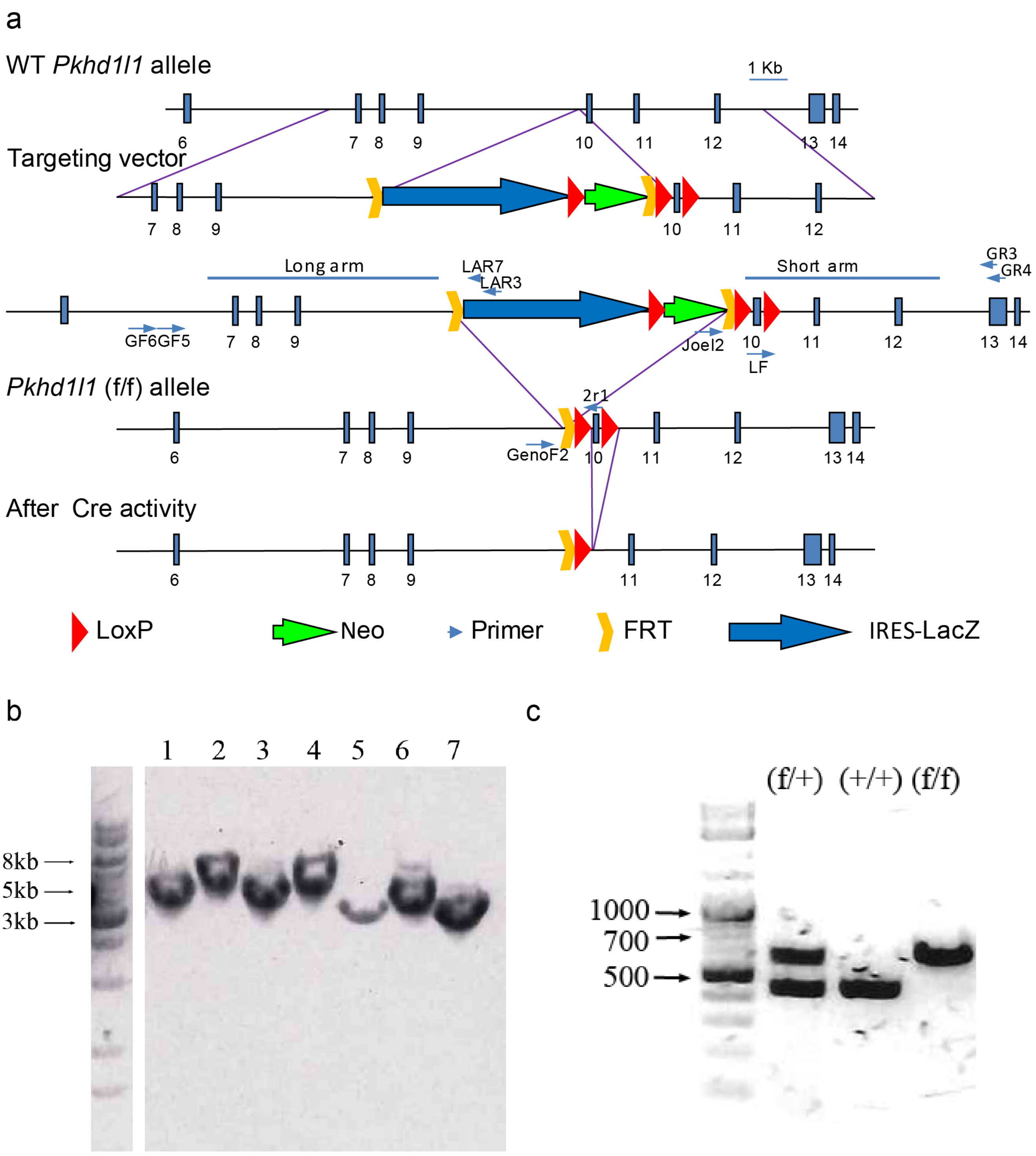

Supplementary Fig. 1. Generation of the PKHD1L1 conditional knockout mouse**. a.** Strategy for generating the *Pkhd1l1* conditional knockout. Two ES clones (Pkhd1l1$^{tm2a(EUCOMM)Hmgu}$ clones HEPD0803_8_E04 and HEPD0803_8_H03) were obtained from the European Conditional Mouse Mutagenesis Program (EUCOMM); map was modified from the EUCOMM website. **b.** The ES cells were confirmed by long template PCR, which produces nucleotides flanking the short and long homologous recombination arms. Seven primer pairs all produced the long template PCR product with the expected size. **c.** Genotyping of wild-type and floxed Pkhd1l1 conditional knockout mice. The floxed allele (f/f) generated a PCR product of 624 bp whereas wild type (+/+) generated a product of 438 bp, as expected. Heterozygotes (f/+) generated both.

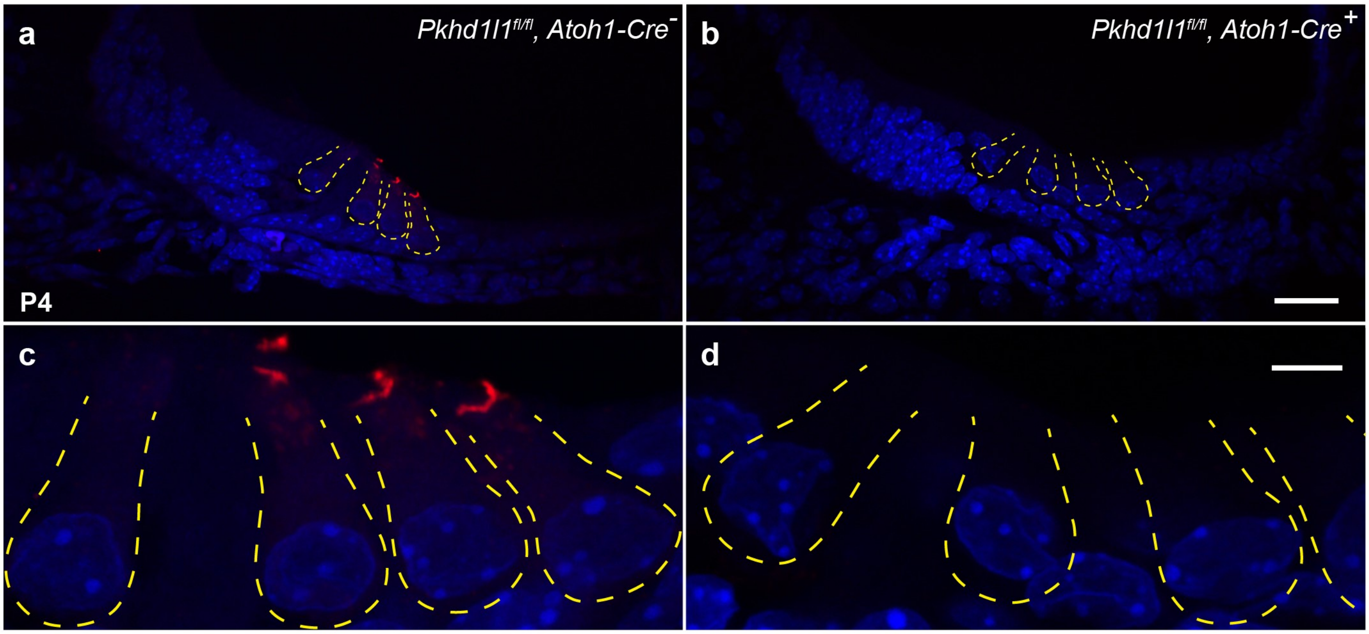

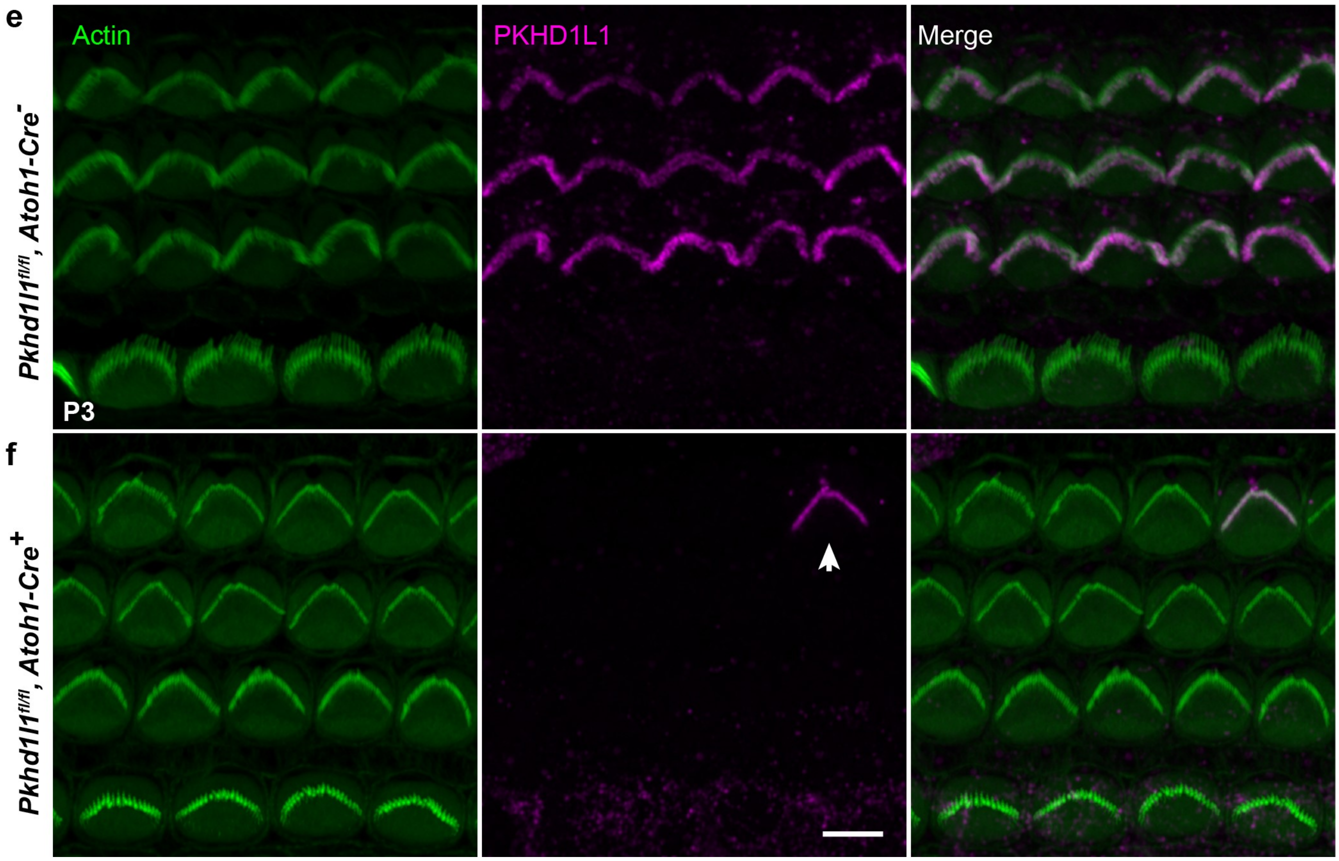

Supplementary Fig. 2. Anti-PKHD1L1 antibody validation. **a–d.** Frozen sections (10-µm thick) and (**e, f**) whole mount organ of Corti explants collected from *Pkhd1l1$^{fl/fl}$, Atoh1-Cre^{-}* (**a, c, e**) and *Pkhd1l1$^{fl/fl}$, Atoh1-Cre^{+}* (**b, d, f**) littermates at P3. Anti-PKHD1L1 (*red*) and DAPI (*blue*) labeling, with yellow dashed lines outlining hair cell bodies. No detectable signal was observed in the bundles or the cell bodies of *Pkhd1l1$^{fl/fl}$, Atoh1-Cre^{+}* hair cells (**b, d**). **e, f.** Anti-PKHD1L1 (*magenta*) and phalloidin labeling (*green*). A rarely observed mosaic effect of the *Atoh1-Cre* enhancer resulted in a PKHD1L1-positive OHC (*white arrow*) in a *Pkhd1l1$^{fl/fl}$, Atoh1-Cre^{+}* mouse (**f**). Scale bars: **a, b,** 20 µm; **c–f,** 5 µm.

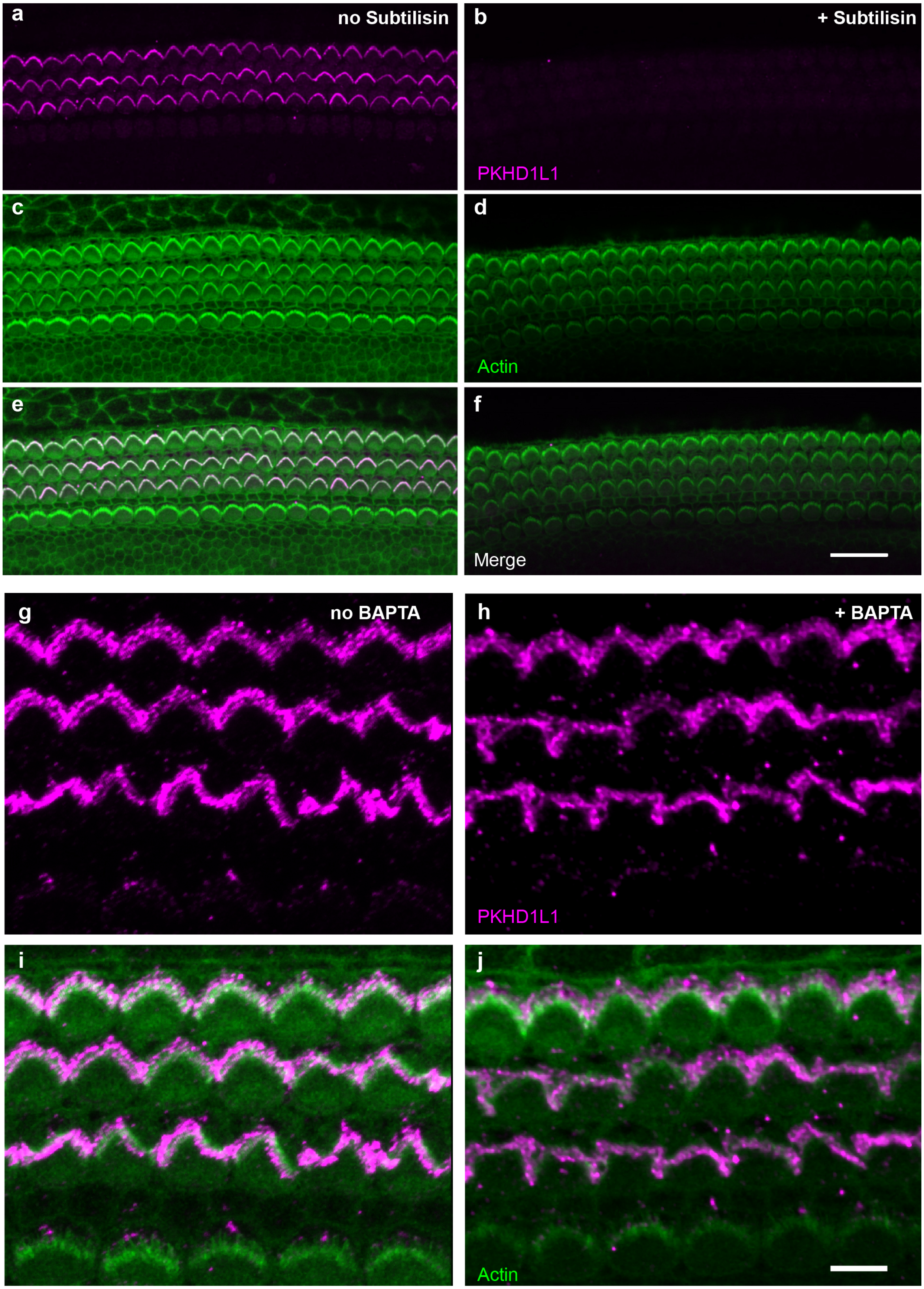

Supplementary Fig. 3. PKHD1L1 labeling is sensitive to subtilisin (**a-f**) but not BAPTA treatment (**g-j**) Subtilisin abolition of anti-PKHD1L1 labeling in hair-cell stereocilia bundles at P4**.** No immunolabeling was observed in OHC bundles of normal *Pkhd1l1*$^{fl/fl}$*, Atoh1-Cre*$^{-}$ mice following subtilisin treatment (**b**, magenta in **f**) (50 µg ml^{-1}, 20 minutes) but not BAPTA (**h**, magenta in **j**) (5 mM, 15 minutes) vs. control, untreated samples (**a**, **g**, magenta in **e, i**). Anti-PKHD1L1 in magenta, phalloidin in green. Scale bars: a-d, 20 µm; g-j, 7 µm.

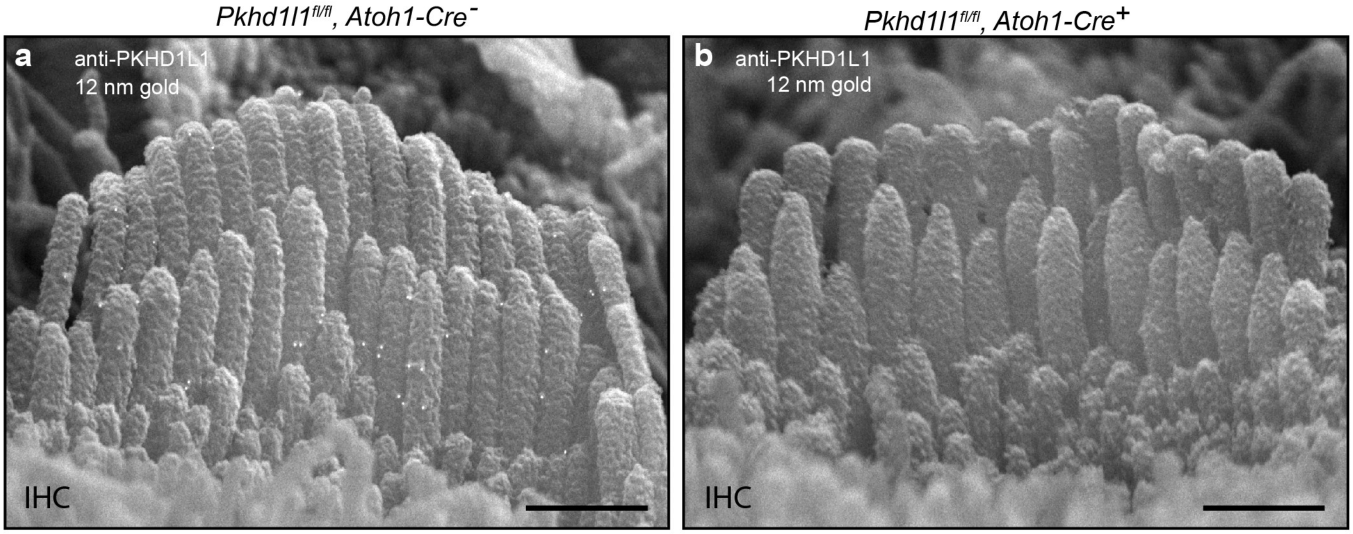

Supplementary Fig. 4. Immunogold SEM localization of PKHD1L1 on the surface of IHC stereocilia (P4). 12-nm gold beads were present on the surface of *Pkhd1l1*$^{fl/fl}$, *Atoh1-Cre*$^{-}$ IHCs (**a**), but not *Pkhd1l1*$^{fl/fl}$, *Atoh1-Cre*$^{+}$ IHCs (**b**). Scale bar, 500 nm.
