# Supplementary material for: PKHD1L1 is a coat protein of hair-cell stereocilia and is required for normal hearing
Source: Nat Commun. 2019 Aug 23;10:3801. doi: 10.1038/s41467-019-11712-w (PMC6707252; doi:10.1038/s41467-019-11712-w)
Supplement: Supplementary file 3 — Reporting Summary [file 41467_2019_11712_MOESM3_ESM.pdf]

nature research

Corresponding author(s): David Corey, Artur Indzhykulian

Last updated by author(s): 05-30-2019

Reporting Summary

Nature Research wishes to improve the reproducibility of the work that we publish. This form provides structure for consistency and transparency in reporting. For further information on Nature Research policies, see Authors & Referees and the Editorial Policy Checklist.

Statistics

For all statistical analyses, confirm that the following items are present in the figure legend, table legend, main text, or Methods section.

n/a	Confirmed	
☐	☒	The exact sample size (*n*) for each experimental group/condition, given as a discrete number and unit of measurement
☐	☒	A statement on whether measurements were taken from distinct samples or whether the same sample was measured repeatedly
☐	☒	The statistical test(s) used AND whether they are one- or two-sided<br>*Only common tests should be described solely by name; describe more complex techniques in the Methods section.*
☐	☒	A description of all covariates tested
☐	☒	A description of any assumptions or corrections, such as tests of normality and adjustment for multiple comparisons
☐	☒	A full description of the statistical parameters including central tendency (e.g. means) or other basic estimates (e.g. regression coefficient) AND variation (e.g. standard deviation) or associated estimates of uncertainty (e.g. confidence intervals)
☐	☒	For null hypothesis testing, the test statistic (e.g. *F*, *t*, *r*) with confidence intervals, effect sizes, degrees of freedom and *P* value noted<br>*Give P values as exact values whenever suitable.*
☒	☐	For Bayesian analysis, information on the choice of priors and Markov chain Monte Carlo settings
☒	☐	For hierarchical and complex designs, identification of the appropriate level for tests and full reporting of outcomes
☒	☐	Estimates of effect sizes (e.g. Cohen's *d*, Pearson's *r*), indicating how they were calculated

Our web collection on statistics for biologists contains articles on many of the points above.

Software and code

Policy information about availability of computer code

Data collection	Confocal imaging data was collected using Olympus FluoView software package; FIB SEM data was collected using FEI Slice and View software package
Data analysis	Data was analyzed using Excel, Prizm, and custom Matlab code as described in the methods section

For manuscripts utilizing custom algorithms or software that are central to the research but not yet described in published literature, software must be made available to editors/reviewers. We strongly encourage code deposition in a community repository (e.g. GitHub). See the Nature Research guidelines for submitting code & software for further information.

Data

Policy information about availability of data

All manuscripts must include a data availability statement. This statement should provide the following information, where applicable:

- Accession codes, unique identifiers, or web links for publicly available datasets
- A list of figures that have associated raw data
- A description of any restrictions on data availability

The datasets generated during and/or analysed during the current study are available from the corresponding author on reasonable request

Field-specific reporting

Please select the one below that is the best fit for your research. If you are not sure, read the appropriate sections before making your selection.

☒ Life sciences ☐ Behavioural & social sciences ☐ Ecological, evolutionary & environmental sciences

Life sciences study design

All studies must disclose on these points even when the disclosure is negative.

Sample size	Sample size calculations were carried out using power analysis, or based on similar studies published from other groups.
Data exclusions	all cases of data exclusions and the exclusion criteria were discussed in the Methods section of the manuscript
Replication	All experiments except for results presented in Figure 5 (FIB-SEM results) were successfully replicated, and represent results from at least three independent experiments. Because the data in Figure 5 are extremely difficult to collect, all 6 cells of the data set were collected from the same sample (inner ear tissue collected from one mouse). The antibodies used in this study were validated using a number of light and electron microscopy techniques, and the Figure 5 immunogold FIB-SEM results are in agreement with the immunogold SEM and immunogold TEM data. Specifically, a number of samples from the same experiment were first studied using TEM, then one was selected for further imaging using FIB-SEM.
Randomization	Animals were randomly allocated to experimental groups in this study. In some cases the genotyping was performed after the experiments were complete and data collected.
Blinding	Investigators were blinded only in experiments with ABR and DPOAE measurements, in which case they were blinded to the genotype of the animals.

Reporting for specific materials, systems and methods

We require information from authors about some types of materials, experimental systems and methods used in many studies. Here, indicate whether each material, system or method listed is relevant to your study. If you are not sure if a list item applies to your research, read the appropriate section before selecting a response.

Materials & experimental systems

n/a	Involved in the study	
☐	☒	Antibodies
☒	☐	Eukaryotic cell lines
☒	☐	Palaeontology
☐	☒	Animals and other organisms
☒	☐	Human research participants
☒	☐	Clinical data

Methods

n/a	Involved in the study	
☒	☐	ChIP-seq
☒	☐	Flow cytometry
☒	☐	MRI-based neuroimaging

Antibodies

Antibodies used	rabbit anti-PKHD1L1, Novus Bio #NBP2-13765; donkey anti-rabbit ThermoFisher #A10042; EM Goat F(ab')2 anti-rabbit IgG:10 nm gold (BB International) #14216, JAX Immunoresearch 12 nm Colloidal Gold AffiniPure Goat Anti-Rabbit IgG (H+L) #111-205-144
Validation	validated using a knock-out mouse model

Animals and other organisms

Policy information about studies involving animals; ARRIVE guidelines recommended for reporting animal research

Laboratory animals	mice on a mixed C57BL/6N/129S4/CBA genetic background
Wild animals	the study did not involve wild animals
Field-collected samples	the study did not involve samples collected in the field
Ethics oversight	the study was approved by HMS IACUC and MEEI Animal Care Committee

Note that full information on the approval of the study protocol must also be provided in the manuscript.
