# Supplementary material for: PKHD1L1 is a coat protein of hair-cell stereocilia and is required for normal hearing
Source: Nat Commun. 2019 Aug 23;10:3801. doi: 10.1038/s41467-019-11712-w (PMC6707252; doi:10.1038/s41467-019-11712-w)
Supplement: Supplementary file 4 — Description of Additional Supplementary Information [file 41467_2019_11712_MOESM4_ESM.pdf]

Description of Additional Supplementary Files

File Name: Supplementary Movie 1
Description: **3D localization of PKHD1L1 in P4 OHCs using immunogold FIB- SEM.** A 3D reconstruction of an anti-PKHD1L1 immuno-gold (10 nm) labeled OHC stereocilia bundle from 387 serial FIB-SEM cross-sections, at 15 nm milling step; *yellow*, gold beads; blue, stereocilia; *grey*, cell body.

File Name: Supplementary Movie 2
Description: **A 3D cumulative distribution map of anti-PKHD1L1 immuno-gold beads from six OHC stereocilia bundles.** A 3D cumulative distribution map of anti-PKHD1L1 immuno-gold beads generated with a custom MATLAB algorithm from six reconstructed OHC stereocilia bundles. A 360 degree rotation shows three rows of stereocilia (*yellow* represents gold beads; *gray triangles* represent the stereociliary surface). Kinocilia (*left, light gray*) were excluded from further analysis due to a low number of observations
